# Gut microbial dysbiosis occurring during pulmonary fungal infection in rats is linked to inflammation and depends on healthy microbiota composition

**DOI:** 10.1128/spectrum.01990-23

**Published:** 2023-08-25

**Authors:** Dusanka Popovic, Jelena Kulas, Dina Tucovic, Aleksandra Popov Aleksandrov, Anastasija Malesevic, Jasmina Glamoclija, Emilija Brdaric, Svetlana Sokovic Bajic, Natasa Golic, Ivana Mirkov, Maja Tolinacki

**Affiliations:** 1 Immunotoxicology Group, Department of Ecology, Institute for Biological Research “Sinisa Stankovic” – National Institute of the Republic of Serbia, University of Belgrade, Belgrade, Serbia; 2 Mycology Laboratory, Department of Plant Physiology, Institute for Biological Research “Sinisa Stankovic” – National Institute of the Republic of Serbia, University of Belgrade, Belgrade, Serbia; 3 Group for Probiotics and Microbiota-Host Interaction, Laboratory for Molecular Microbiology, Institute of Molecular Genetics and Genetic Engineering, University of Belgrade, Belgrade, Serbia; University of Minho, Braga, Portugal

**Keywords:** fungal lung infection, gastrointestinal microbiota, lung microbiota, lung-gut axis, rats

## Abstract

While the effect of gut microbiota and/or inflammation on a distant body site, including the lungs (gut–lung axis), has been well characterized, data about the influence of lung microbiota and lung inflammation on gut homeostasis (lung–gut axis) are scarce. Using a well-characterized model of pulmonary infection with the fungus *Aspergillus fumigatus*, we investigated alterations in the lung and gut microbiota by next-generation sequencing of the V3–V4 regions of total bacterial DNA. Pulmonary inflammation due to the fungus *A. fumigatus* caused bacterial dysbiosis in both lungs and gut, but with different characteristics. While increased alpha diversity and unchanged bacterial composition were noted in the lungs, dysbiosis in the gut was characterized by decreased alpha diversity indices and modified bacterial composition. The altered homeostasis in the lungs allows the immigration of new bacterial species of which 41.8% were found in the feces, indicating that some degree of bacterial migration from the gut to the lungs occurs. On the contrary, the dysbiosis occurring in the gut during pulmonary infection was a consequence of the local activity of the immune system. In addition, the alteration of gut microbiota in response to pulmonary infection depends on the bacterial composition before infection, as no changes in gut bacterial microbiota were detected in a rat strain with diverse gut bacteria. The data presented support the existence of the lung–gut axis and provide additional insight into this mechanism.

**IMPORTANCE:**

Data regarding the impact of lung inflammation and lung microbiota on GIT are scarce, and the mechanisms of this interaction are still unknown. Using a well-characterized model of pulmonary infection caused by the opportunistic fungus *Aspergillus fumigatus*, we observed bacterial dysbiosis in both the lungs and gut that supports the existence of the lung–gut axis.

## INTRODUCTION

Bacteria inhabit every part of the human body, but most of them are found in the gut. Gut microbiota are responsible for many functions, including nutrient metabolism, immunomodulation, maintenance of host physiology, and protection against pathogen overgrowth (1). To date, numerous scientific studies confirm the important role of gut bacteria in health and disease. This microbial community impacts not only local immunity but also a distant body site, such as the lungs. Disturbances in gut bacterial composition have been linked to asthma (2), chronic obstructive pulmonary disease (3), cystic fibrosis (4), and lung cancer (5). Furthermore, pulmonary involvement was noted in inflammatory gastrointestinal disease characterized by microbial dysbiosis (6), supporting the existence of a gut–lung axis. The gut bacterial microbiota or some of their constituents impact the immune response in the lungs against viruses (7 – 9), bacteria (10 – 13), fungi (14), and allergic airway inflammation (15) mainly through the effect of the gut microbiota (or their metabolites) on the immune cell activity.

While the gut–lung axis is well characterized, the influence of the lung microbiota as well as lung inflammation on gut homeostasis has attracted much more attention in recent years. The first indication of the lung–gut axis was a higher prevalence (compared to healthy subjects) of gastrointestinal symptoms in patients with asthma (16) and chronic obstructive pulmonary disease (17). The existence of gastrointestinal symptoms in patients with pulmonary virus infection has also been documented (18). Gastrointestinal symptoms (abdominal pain, nausea, vomiting, and diarrhea) were noted in 11.6% of children with influenza infection (18), and a later study showed a decrease in alpha diversity in the feces of influenza-infected patients compared to healthy controls (19). Fecal bacterial samples from patients with COVID-19 infection were shown to cluster separately from those in healthy controls as well, but in the majority of these patients, SARS-Cov-2 could be detected in the feces (20). Experimental studies in mice confirmed the occurrence of gut dysbiosis following respiratory influenza virus infection (21 – 25) and respiratory syncytial virus infection (24), despite the fact that the virus has not been detected in the gut (21, 22, 24, 25). It has been shown that the alteration of gut microbiota is a consequence of infection with live virus particles, as administration of an attenuated influenza vaccine had no effect on the microbiota (24).

Bacterial dysbiosis in the gut also occurs following pulmonary bacterial infection. A decrease in alpha diversity indices and differential relative abundance of fecal microbiota were noted in patients with pulmonary tuberculosis (26, 27) and in mice infected with *Mycobacterium tuberculosis* (28) and *Klebsiella pneumoniae* (29). Even administration of the major component of the outer membrane of Gram-negative bacteria, lipopolysaccharide, to the lungs caused gut bacteria dysbiosis (30).

In addition to pulmonary infections caused by viruses or bacteria, alteration of the gut microbiota was noted in mice exposed to hyperoxia (31) and in patients with lung cancer (compared to healthy individuals) (32) indicating that pulmonary inflammation/injury affects the gut microbiota regardless of its origin. Despite a growing body of evidence for interaction between the lungs and gut, there is still a lot of work to be done to understand this crosstalk. There are virtually no data regarding gut microbiota changes during pulmonary infection caused by fungi. Our previous study showed an alteration in immune-mediated homeostasis of the gut in a rat model of sublethal pulmonary infection with *A. fumigatus* (33). Using the same experimental model of infection in Dark Agouti (DA) rat strain, we aimed to investigate changes in the lung and gut microbiota by next-generation sequencing of the V3–V4 regions of total bacterial DNA in these two organs. Possible mechanisms of lung–gut communication were also investigated. In addition, to examine whether gut dysbiosis is a general characteristic during pulmonary fungal infection, we analyzed feces from infected Albino Oxford (AO) rats, a strain that develop quantitatively different immune response to fungus *A. fumigatus* (34) and whose gut microbiota was previously shown to respond differently to oral cadmium administration (35) compared to DA rats.

## MATERIALS AND METHODS

### Rats

Male DA rats used in this study were 10 to 12 weeks old, bred, and conventionally housed (12 h light/dark cycle, ambient temperature of 22 ± 2°C and 60% relative humidity, unlimited access to standard rodent pellet and water) at the Institute for Biological Research “Sinisa Stankovic” (Belgrade, Serbia). Eight individuals were assigned per group in two independent experiments. All animal procedures complied with the EEC Directive (86/609/EEC) on the protection of animals used for experimental and other scientific purposes and were approved by the Veterinary Directorate, Ministry of Agriculture, Forestry and Water Management (No 323–07-12685/2020-05). Animals were housed in a common animal housing room and allowed to acclimate for 1 wk before the experimental procedure. Four individuals were randomly assigned per group in two independent experiments (in total eight individuals per group). In some experiments, male AO rats of similar age were used. Fungal culture conditions and experimental infection of animals were done as previously described (33, 34). In brief, a human isolate of *Aspergillus fumigatus* (obtained from the Institute of Public Health of Serbia “Dr. Milan Jovanovic-Batut”) was grown on Sabouraud maltose agar (SMA, Torlak, Belgrade, Serbia) for 7 days to produce conidia, which were thereafter collected by flooding the surface of agar slants with pyrogen-free sterile physiological saline (Hemofarm, Vrsac, Serbia). Rats were injected with 10^7^ conidia/mL in 0.05 mL of saline [into the trachea of anesthetized rats (Zoletil 100, Virbac, Carros, France)]. The control group (sham-infected rats) received saline only. No animal died during the experiment.

### Sacrificing of animals, tissue sample collection, and homogenate preparation

Animals were euthanized at days 1 and 3 post-infection (p. i.) by intraperitoneal injection of 15 mg/kg b.w. of Zoletil 100 (Virbac, Carros, France). Lung samples, duodenum (5 cm immediately below the pyloro-duodenal junction)), ileum (3 cm above the ileo-cecal junction), cecum, and colon were aseptically removed. Intestinal content (collected by extrusion), intestinal tissue (free of content and the lumen was washed with ice-cold non-pyrogenic physiological saline), and the lungs were used for bacterial DNA extraction. Preliminary examination of bacterial dysbiosis in various segments of the gastrointestinal tract (GIT) was done using denaturing gradient gel electrophoresis (DGGE) analysis (36).

Segments of intestine were washed through the lumen with ice-cold non-pyrogenic physiological saline, weighed, snap frozen in liquid nitrogen, and stored at –80°C until use. Tissue samples were homogenized as previously described (33), and obtained supernatants were used for cytokine measurements.

### Bacterial DNA extraction and sequencing

Total genomic DNA extraction from frozen tissue samples (randomly selected four samples from each experimental group) was performed with ZR Tissue DNA MiniPrep™ Kit (Zymo Research Corp., Irvine, CA USA), according to the manufacturer’s instruction, and the concentration of isolated DNA was measured on Qubit fluorometer (ThermoFisher/Invitrogen, Waltham, MA USA). Samples were diluted to the concentration of 12 ng/µL in 20 µL of final volume and sent to Novogene Company (Cambridge, United Kingdom) for library preparation and 16S rRNA amplicon sequencing of the V3–V4 hypervariable region using 341 (forward, 5’–CCTAYGGGRBGCASCAG–3’) and 806 (reverse, 5’–GGACTACNNGGGTATCTAAT– 3’) primers. Paired-end sequencing was performed on an Illumina NovaSeq 6,000 platform. The quality of sequencing was analyzed using the rarefaction method. For each sample, sufficient depth was achieved during sequencing, and the coverage index for each sample was greater than 0.991, which is sufficient to reflect the exact composition of the microbiota (37). Paired-end reads were merged using FLASH (V1.2.7) (38). To obtain high-quality clean tags, quality filtering on the raw tags was performed under specific filtering conditions (39) according to the QIIME (40). For each representative sequence, QIIME Version 1.7.0 (41) in the Mothur method was performed against the SSUrRNA database of SILVA Database (42) for species annotation at each taxonomic rank. Alpha diversity and beta diversity analyses were performed based on the normalized operational taxonomic unit (OTU) data. Alpha diversity (expressed through three indices, including Observed species, Shannon, and Chao1 index) and beta diversity calculations were done in QIIME and displayed with R software (Version 2.15.3), performed by Novogene Technology Co. Ltd. Results were visualized using principal coordinate analysis (PCoA), and expressed through ANOSIM and Adonis. Linear discriminant analysis effect size (LEfSe) was done using the Galaxy platform (43).

### Determination of hematologic parameters

Blood was withdrawn from the abdominal artery for the determination of leukocyte counts, hematologic parameters, and plasma isolation. Differential leukocyte counts and hematological parameters were determined automatically by Siemens ADVIA 120 flow cytometer (Terytown, New York, USA) using commercially available reagents. Following centrifugation, plasma was collected for interleukin-6 (IL-6), IL-17, and tumor necrosis factor (TNF) measurements.

### Enzyme-linked immunosorbent assay (ELISA)

Cytokine concentrations in tissue homogenates and plasma were determined using commercially available ELISA sets for IL-10, IL-6, TNF, IL-1β (R&D Systems, Minneapolis, USA), IL-17, and IFN-γ (eBioscience Inc., San Diego, CA, USA) according to the manufacturer’s instructions. The standard curve generated using known amounts of the respective set provided recombinant cytokines was used to calculate cytokine titers.

### Data display and statistical analysis

Results are expressed as mean ± standard deviation (SD). The Statistica 7.0 (StatSoft Inc., Tulsa, OK) software was used for statistical analysis. Multiple comparisons between groups were done using one-way ANOVA followed by Tukey’s test and the probability level less than 0.05 was considered significant.

## RESULTS

### Dysbiosis occurs in the duodenum and fecal samples

Since, according to the literature, different segments of the gastrointestinal tract are used for the examination of microbiota, we first analyzed in which segment the most prominent changes in the microbiota occur during fungal infection. Using the DGGE method, we examined bacteria attached to the wall of GIT (bacteria that remained after rinsing off the luminal content) and luminal content from the duodenum, ileum, cecum, and colon (Fig. S1; Table S1). A decrease in the number of DGGE bands was noted in the duodenum (at day 1 p.i. in the wall) and feces at both time points examined. No statistically significant changes were observed in the ileum and cecum, nor in the lumen of the duodenum or in bacteria attached to the wall of the colon. As the highest number of DGGE bands and continuous decline in the number of bands were noted in feces, and having in mind the easy availability of fecal samples, we decided to use these samples for next-generation sequencing.

### During pulmonary infection, bacterial dysbiosis occurs in both the lungs and the GIT

**Fig 1:**
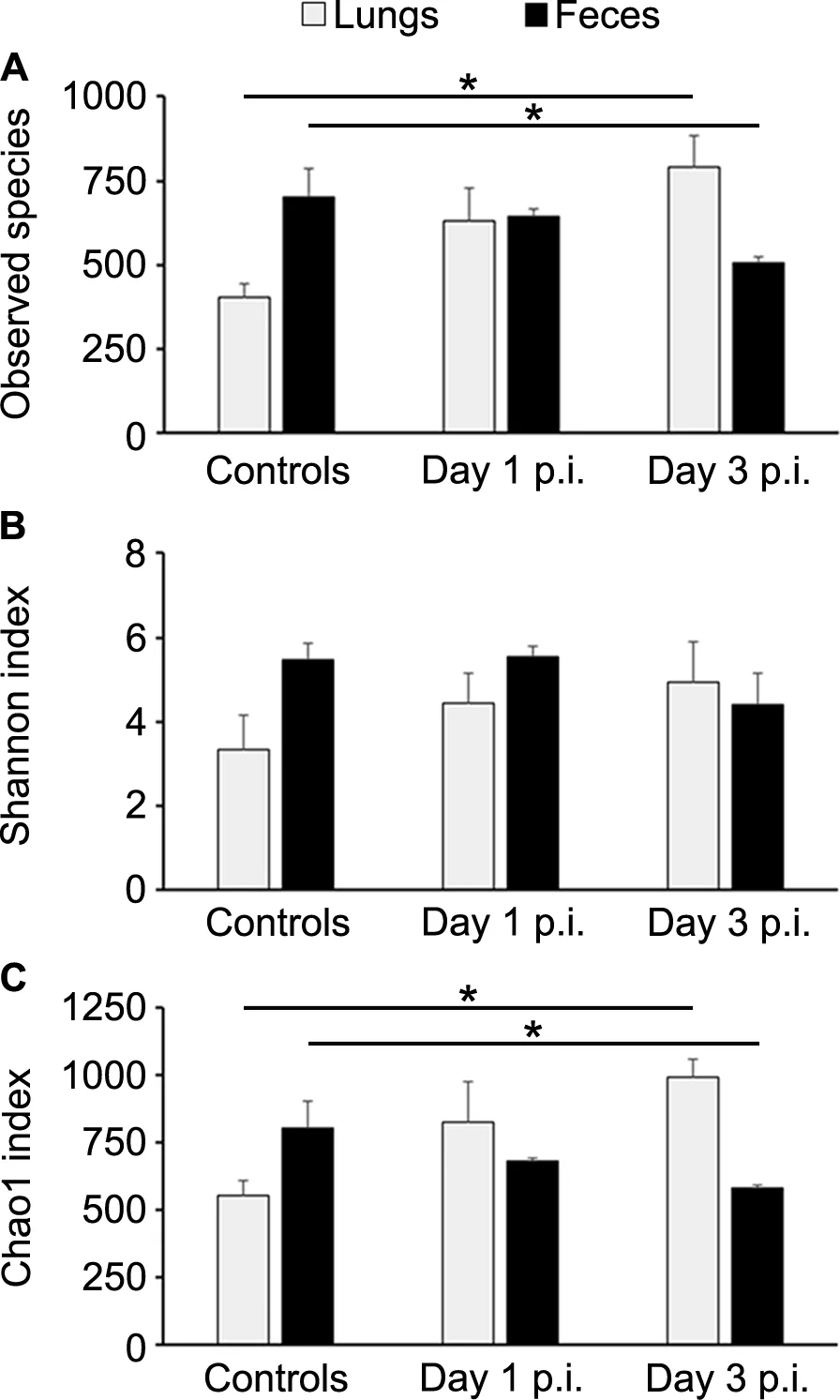
Alpha diversity in lungs and feces during pulmonary infection with *A. fumigatus*. (**A**) Observed species. (**B**) Shannon index. (**C**) Chao1 index. Results are expressed as mean ± SD. Statistically significant at * *P* < 0.05 *vs* controls.

As expected, higher indices of alpha diversity (observed species, Shannon and Chao1 indexes) were noted in the fecal samples compared to the lungs (Fig. 1) in controls. During pulmonary infection, dysbiosis was detected in both samples but, while in the lung microbiota, an increase in number of observed species (Fig. 1A) and Chao1 index (Fig. 1C) were detected at day 3 p.i., and, while in the fecal samples, decreased alpha diversity (number of observed species and Chao1 index) occurred at the same time point. Examination of bacterial composition in the lungs revealed no differences between controls and infected animals (ANOSIM R = −0.096, *P* = 0.639 and R = 0.074, *P* = 0.272 for day 1 and day 3 p.i., respectively). In contrast to that, in the fecal samples, a significant difference in composition was noted between controls and day 1 p.i. (ANOSIM R = 0.593, *P* = 0.029) but not between controls and day 3 p.i. (ANOSIM R = 0.315, *P* = 0.08).

**Fig 2:**
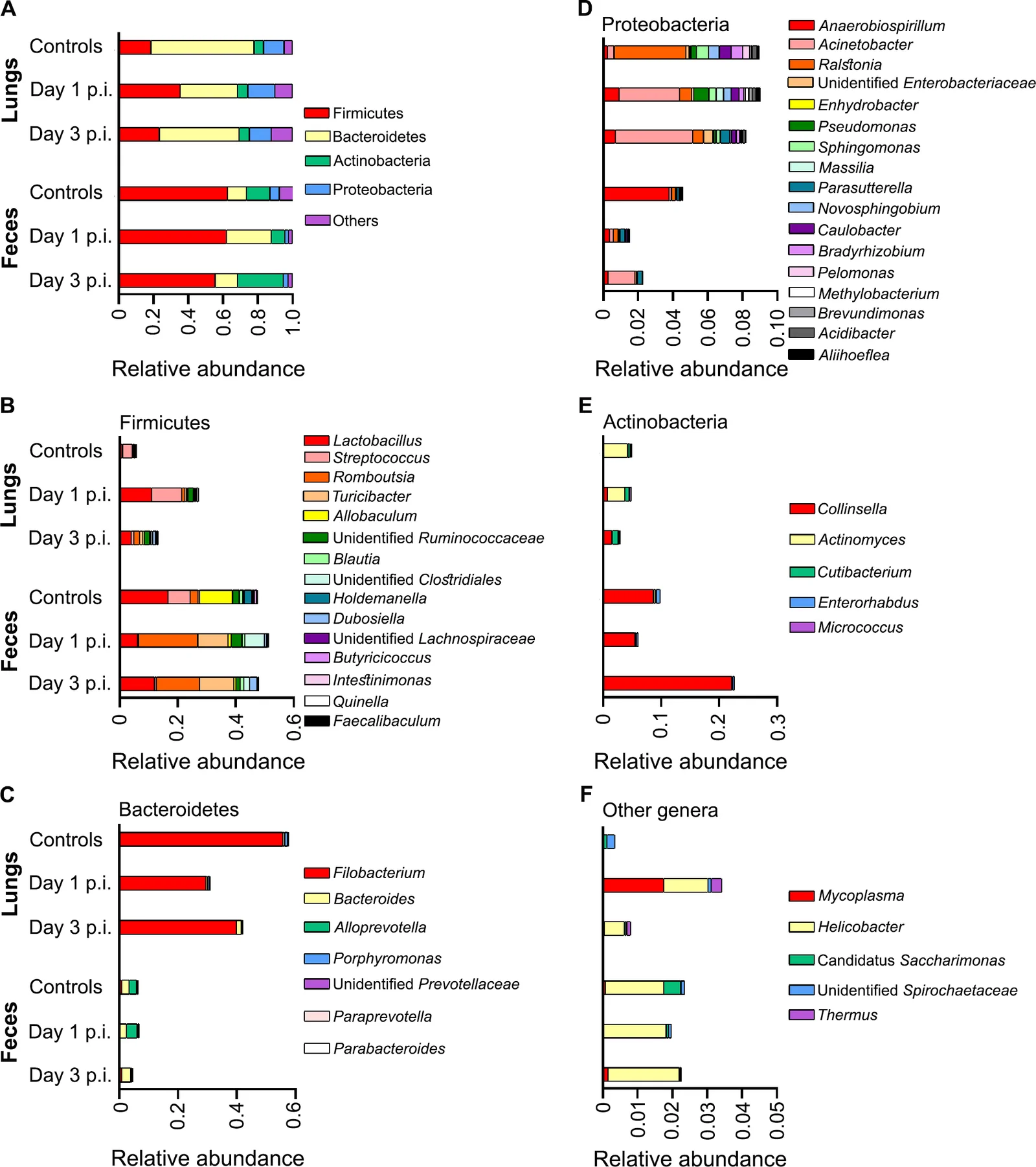
Relative abundance of OTUs in lungs and feces during pulmonary infection with *Aspergillus fumigatus*. (**A**) Phylum level. Abundance of representatives of phylum Firmicutes (**B**), Bacteroidetes (**C**), Proteobacteria (**D**), Actinobacteria (**E**), and other genera (**F**).

Examination of relative abundances at the phylum level revealed higher abundance of Firmicutes and Actinobacteria in the fecal samples and of Bacteroidetes and Proteobacteria in the lungs (Fig. 2A). No statistically significant changes were detected in either sample during infection. Furthermore, we analyzed changes in the relative abundance of genera (with relative abundance above 0.001 in either experimental group). Within phylum Firmicutes, 15 genera had relative abundance above the selected threshold (Fig. 2B). During pulmonary infection, an increase in *Romboutsia* was noted in the lungs at day 3 p.i. (*P* = 0.0034) and in the feces at both time points (*P* < 0.05). An increase in the lungs but a decrease in the fecal samples at both time points were observed for genus *Butyricicoccus* (*P* < 0.05). An increase in the relative abundance of genera *Turicibacter* (at day 3 p.i., *P* = 0.0155), unidentified *Ruminococcaceae* (at both time points, *P* < 0.001), and *Holdemanella* (at both time points, *P* < 0.01) has been detected in the lungs solely. Following pulmonary infection, decreased abundance of *Streptococcus* (at both time points, *P* < 0.05), *Allobaculum* (at both time points, *P* < 0.05), and *Quinella* (at day 3 p.i., *P* = 0.0392), but an increased unidentified *Clostridiales* (at day 1 p.i., *P* = 0.0138) and *Dubosiella* (at day 3 p.i., *P* = 0.0128), occurs in feces. From the representatives of phylum Bacteroidetes (seven genera) (Fig. 2C), solely *Paraprevotella* was increased at day 1 p.i. (*P* = 0.0084) in the fecal samples. Within phylum Proteobacteria, 16 genera were analyzed (Fig. 2D). Pulmonary infection resulted in an increase of *Anaerobiospirillum* in the lungs at day 1 p.i. (*P* = 0.0335) and *Parasutterella* (at day 3 p.i., *P* = 0.045) but a decrease in *Ralstonia* (at both time points, *P* < 0.01). In the fecal samples, solely a decrease in abundance of *Novosphingobium* at day 3 p.i. was detected (*P* = 0.037). In phylum Actinobacteria (Fig. 2E), solely a decrease in *Enterorhabdus* at both time points examined in feces was noted (*P* < 0.05). Regarding representatives of the other phyla (Fig. 2F), a decrease in abundance of Candidatus *Saccharimonas* in the lungs at day 1 p.i. (*P* = 0.0217) and feces at day 3 p.i. (*P* = 0.0467) was observed.

### Microbiota differs between the lungs and the feces in healthy animals and during pulmonary infection

**Fig 3:**
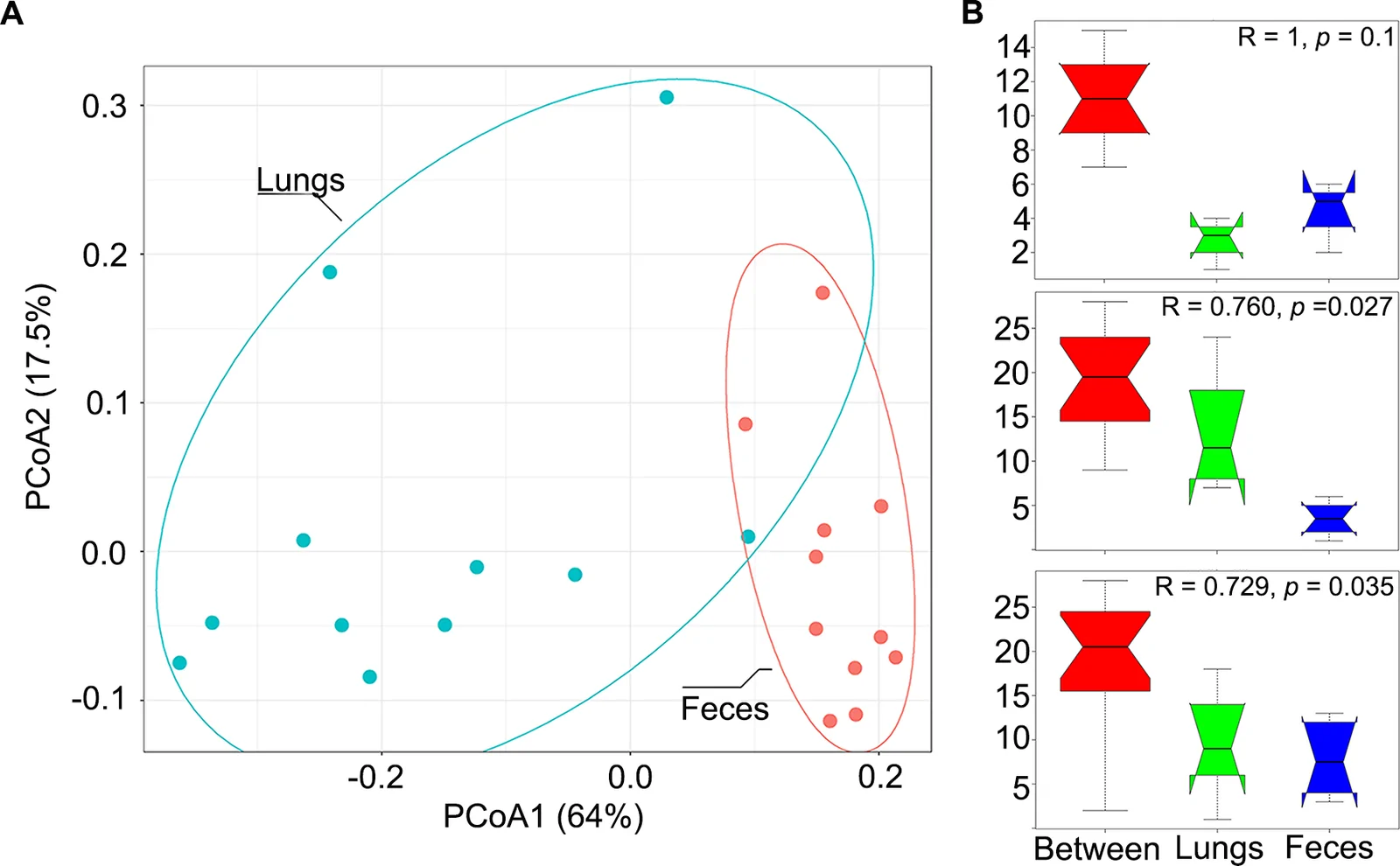
Comparison of microbial composition in lungs and feces. (**A**) PCoA. (**B**) ANOSIM in controls, at day 1 and day 3 p.i.

Since it is assumed that the mechanisms of interaction between the lungs and the GIT include translocation of microbiota from the lungs to the GIT (aspiration of bronchus content) and from the GIT to the lungs (probably *via* blood or lymph due to epithelial damage in the GIT), and having in mind that rats are coprophagous animals (potentially allowing fecal bacteria to enter the lungs by microaspiration), we compared the microbial community composition in the lungs and the feces. Principal coordinate analysis (PCoA) revealed an obvious separation of microbial composition in the lungs and the gut (Fig. 3A), which was confirmed by the analysis of similarity (although the difference was not statistically significant for the control group) (Fig. 3B). ADONIS indicated that 52.1% in controls (*P* = 0.1), 44.3% at day 1 p.i. (*P* = 0.031), and 39.0% at day 3 p.i. (*P* = 0.032) OTUs were different between the lungs and the feces.

Analysis of the detected OTUs revealed a total of 1,581 OTUs in the lungs and 837 in the feces, of which 190 OTUs were detected in both organs in all experimental groups. During infection, 923 OTUs occur *de novo* in the lungs (386 OTUs that are detected in the fecal samples and 537 OTUs undetectable in the feces).

### The GIT dysbiosis during pulmonary infection was not the result of a systemic reaction

Another possible mechanism of lung–gut interaction is alteration in the systemic circulation. To investigate this possibility, we analyzed basic hematological parameters in peripheral blood (including total leukocyte, neutrophil, lymphocyte, monocyte, eosinophil, basophil, red blood cell, hemoglobin, hematocrit, and platelet counts), as well as plasma IL-6, TNF and IL-17 levels. No statistically significant changes were detected in the systemic circulation during infection (Table S2), indicating that inflammation was confined to mucosal surfaces.

### Inflammation in the GIT coincides with dysbiosis

**Fig 4:**
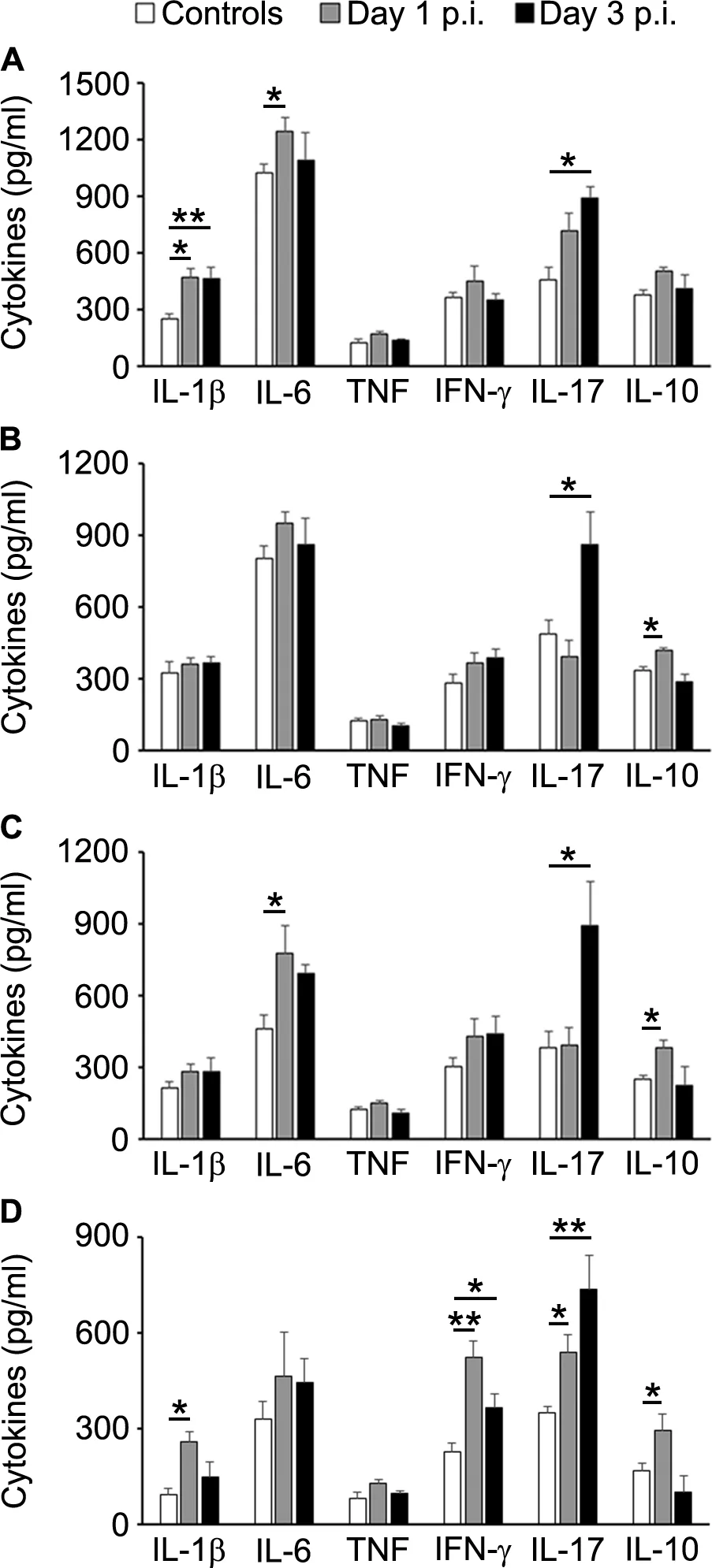
Inflammation in gastrointestinal tract during pulmonary infection. (**A**) Colon, (**B**) Cecum, (**C**) Ileum, (**D**) Duodenum. Results are expressed as mean ± SD. Statistically significant at *P < 0.05 and **P < 0.01 *vs* controls.

Having in mind data indicating inflammation in the GIT that occurs during pulmonary infection as a causative agent of dysbiosis (21), we next measured cytokine content in the colon. Increased levels of IL-1β (at both time points), IL-6 (at day 1 p.i.), and IL-17 (at day 3 p.i.) were detected in infected animals compared to controls (Fig. 4A). In addition to the colon, a higher cytokine content in infected compared to control animals was noted in other segments of the GIT (Fig. 4B through D). In the cecum, IL-17 (at day 3 p.i.) and IL-10 (at day 1 p.i.) were increased (Fig. 4B). A higher content of IL-6 (at day 1 p.i.), IL-17 (at day 3 p.i.), and IL-10 (at day 1 p.i.) was noted in ileum (Fig. 4C), while in the duodenum, an increase in IL-1β and IL-10 was evident at day 1 p.i., and IFN-γ and IL-17 at both time points were examined (Fig. 4D).

### The occurrence of GIT dysbiosis could depend on microbial composition

**Fig 5:**
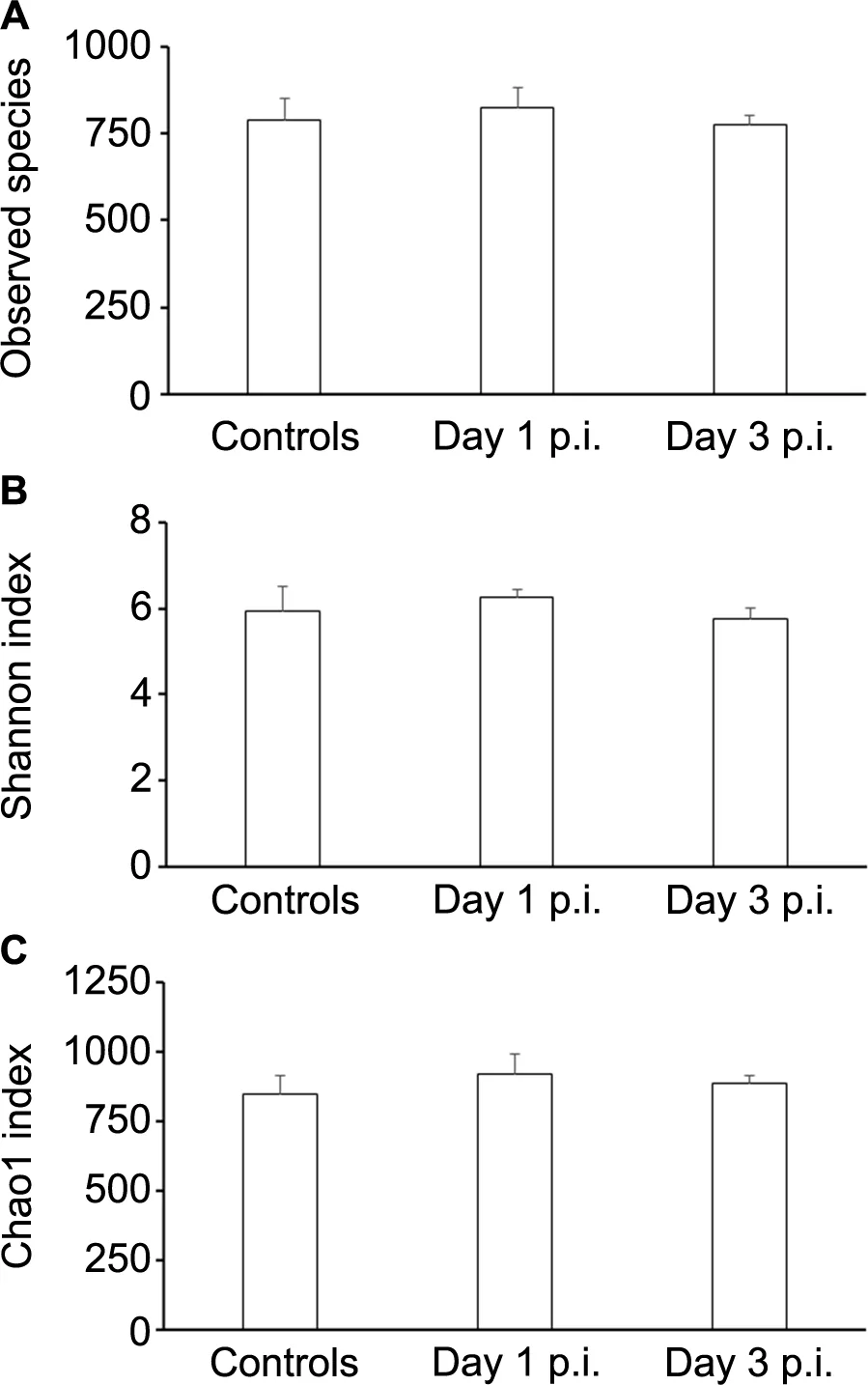
Alpha diversity of fecal microbiota in AO rats during pulmonary infection. (**A**) Observed species. (**B**) Shannon and (**C**) Chao1 indexes. Results are expressed as mean ± SD.

To examine whether this is a general characteristic during pulmonary infection, we next analyzed feces from infected AO rats, a strain in which pulmonary inflammation caused by the fungus *A. fumigatus* is also described (34). No differences in number of observed species (Fig. 5A), Shannon index (Fig. 5B), and Chao 1 (Fig. 5C) index were noted in feces during lung infection in this rat strain. In addition, the analysis of similarity revealed no differences in microbial composition between controls and infected animals at day 1 p.i. (R = 0.185, *P* = 0.3) or day 3 p.i. (R = −0.259, *P* = 0.92).

**Fig 6:**
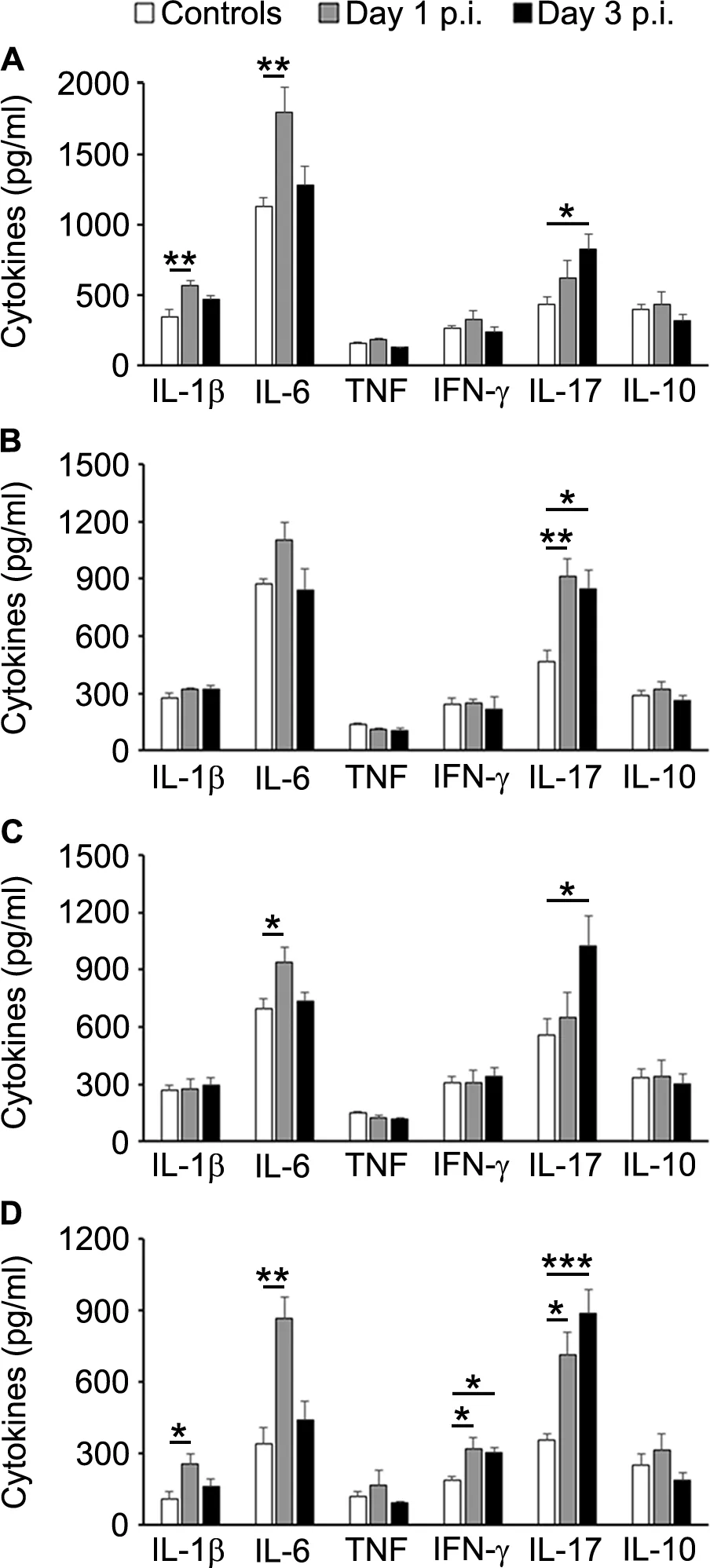
Inflammation in gastrointestinal tract in AO rats during pulmonary infection. (**A**) Colon, (**B**) Cecum, (**C**) Ileum, (**D**) Duodenum. Results are expressed as mean ± SD. Statistically significant at *P < 0.05, **P < 0.01 and ***P < 0.001 *vs* controls.

Following pulmonary infection, increased IL-1β (at day 1 p.i.), IL-6 (at day 1 p.i.) and IL-17 (at day 3 p.i.) were also detected in the colon of this rat strain (Fig. 6A). Similar to the DA rats, inflammation was detected in other segments of the GIT (Fig. 6B through D). A higher IL-1β content was noted in the duodenum at day 1 p.i. and, at the same time point, an increase in IL-6 content was observed in the duodenum and ileum. Levels of IFN-γ and IL-17 were higher in infected animals compared to controls at both time points in the duodenum, while IL-17 was also increased in the ileum (at day 3 p.i.) and the cecum (at both time points).

**Fig 7:**
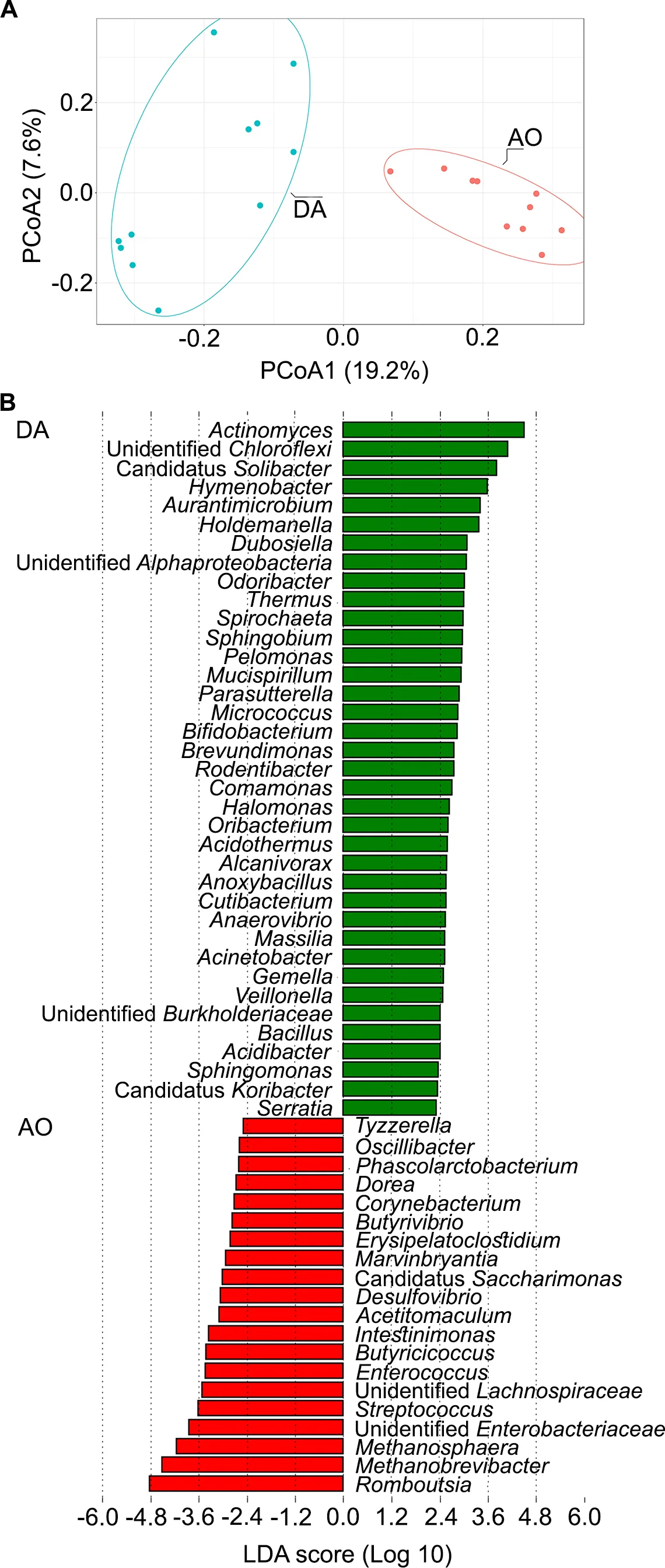
PCoA (**A**) and LEfSe (**B**) analyses of fecal samples in healthy DA and AO rats.

As inflammation was noted in the intestine of AO rats, but without effect on bacterial microbiota, we assumed that differential microbial composition in healthy (untreated) DA and AO rats could be responsible for distinct response to pulmonary inflammation (dysbiosis in DA but without effect in AO rats). PCoA showed clear separation between the fecal samples from DA and AO rats (Fig. 7A), which was confirmed by the analysis of similarity (R = 0.531, *P* = 0.001). In addition, LEfSe analysis revealed a total of 57 genera that were differently abundant in DA (37) and AO (20) rats (Fig. 7B). In DA rats, 40.5% of the genera belong to the phylum Proteobacteria (class *Alphaproteobacteria* 10.8%, *Betaproteobacteria* 13.5% and *Gammaproteobacteria* 16.2%), while in AO rats, 70.0% of the genera belong to Firmicutes (class *Clostridia* 50%, *Bacilli* 10%, *Negativicutes* 5%, and *Erysipelotrichia* 5%).

## DISCUSSION

In this study, the alteration of the bacterial microbiota in the lungs and gut after pulmonary *A. fumigatus* infection was investigated. The results indicate that pulmonary infection caused changes in alpha diversity in both organs, but the microbial composition was altered only in the feces. Gut dysbiosis in DA rats was a consequence of inflammation, which was found in all intestinal segments, but was the most pronounced in the duodenum. Furthermore, alteration of the gut microbiota during pulmonary infection is not a general rule, as it was not detected in AO rats (a strain in which intestinal inflammation was noted).

Microbial composition differs throughout the gastrointestinal tract (from the duodenum to colon) as well as between the mucosa and gut lumen (44). Using the DGGE method, we have shown that during pulmonary infection caused by the fungus, a decrease in bacterial microbiota occurs in the mucosa of the duodenum (at day 1 p.i.) and in the lumen of the colon (at both time points studied). A decrease in the number of DGGE bands in the whole duodenum (lumen and mucosa) following *A. fumigatus* infection has been noted previously (35), and the current results suggest that the dysbiosis in the duodenum was a consequence of the altered microbiota in the mucosa. The absence of changes in DGGE profiles in other compartments studied does not mean that there are no changes in these compartments, as the DGGE method has shortcomings (such as limited number of bands that can be detected, not all bacterial taxa are captured when universal bacterial primers are used, fewer bands detected in contaminated samples, etc.,) (45), but it suggests that the most profound changes during infection occurred in the colon content (i.e., feces).

In pulmonary infection, bacterial dysbiosis occurs not only in the lungs but also in the gut. While increased alpha diversity indices (observed species and Chao1 index) and unchanged beta diversity were found in the lungs, a decreased number of observed species and Chao1 index concomitantly with altered bacterial composition were detected in fecal samples after pulmonary infection with the fungus. Examination of lung-gut crosstalk is a relatively new field of investigation, and, to our knowledge, papers describing lung and gut microbiota interactions in the settings of pulmonary injury/inflammation are scarce. Ashley et al. (31) have shown that lung injury following acute hyperoxia results in a change of bacterial community composition in both the lungs and gut, with no effect on the total bacterial burden. In contrast, no effect on lung microbiota, but reduced community richness and altered bacterial composition in the small intestine in animals infected with influenza virus (25), or an increase in bacterial load in the cecum of mice after intratracheal instillation of LPS (30), were noted. There are also data showing the absence of gut microbiota alteration in the settings of pulmonary inflammation in a mouse model of asthma in which the lung bacterial composition was changed (46). This discrepancy in results could probably be explained by the inflammation/infection model used.

Pulmonary infection has differential effects on the relative abundance of genera in the lungs and gut. Our data indicate that there are genera whose relative abundance has been changed solely in the lungs (*Turicibacter, unidentified Ruminococcaceae*, *Holdemanella*, *Anaerobiospirillum*, *Parasutterella*, *Ralstonia*) or in the feces (*Streptococcus*, *Allobaculum*, *Quinella*, unidentified *Clostridiales*, *Dubosiella*, *Paraprevotella*, *Novosphingobium, Enterorhabdus*), and genera whose relative abundance has increased in the lungs but decreased in the gut (*Butyricicoccus*). Differential effects of lung inflammation on the microbiota in the lungs (a decrease in *Clostridia* and *Bacteroidia* class and an increase in *Staphylococcus* family) and the gut (depletion of Firmicutes phylum and *Ruminococcaceae* family and enrichment of phylum Bacteroidetes) have been documented in the model of acute hyperoxia (31). Results that we obtained further support the existence of a connection between the lungs and the gut, but the local microenvironment probably determines the effect of inflammation on the bacterial microbiota in these two compartments.

According to one theory of lung–gut crosstalk, the interaction between the lung and gut microbiota might occur through the migration of bacteria from the lungs to the gut and from the gut to the lungs (swallowing of bronchial secret, microaspiration, and disruption of the epithelial barrier through which bacteria can enter the blood or lymph, escape of live bacteria from macrophages, etc.,) (47, 48). Our results show that beta diversity differs between the lung and gut even in healthy animals, which is in line with previous findings (49, 50). Noted difference persists after infection, although it seems that the lung and gut microbiota became more similar during infection judging by a decrease in ADONIS *R^2^* (from 0.521 in healthy animals to 0.39 in infected animals at day 3 p.i.). This could be explained by the fact that the number of genera increases in the lungs and decreases in gut during infection, probably causing convergence of bacteria in these two organs. It seems that inflammation noted in the lungs after the fungal infection (51) disturbs regulatory mechanisms responsible for the maintenance of the lung bacterial community, affecting the balance between immigration and elimination of bacteria from the lungs (52). Of 923 OTUs characteristic for the lungs of infected animals, 41.8% were detected in the fecal samples, suggesting the existence of some form of bacterial migration from the gut to the lungs (probably by microaspiration in these experimental settings), but 58.2% OTUs were not present in the feces. In general, bacterial migration from the gut to the lungs (and vice versa) is probably present to some extent but could not completely explain the mechanisms of gut–lung interaction.

Although correlations between systemic cytokine levels and bacterial abundance in feces were noted during pulmonary influenza infection (23) and in patients with lung cancer (32), no changes in peripheral blood and plasma cytokine content were detected in our experimental model. Results obtained indicate that pulmonary infection with *A. fumigatus* does not have systemic character and are in accordance with previously published data showing no effect of pulmonary infection on spleen cell activities (33). This suggests that the microbial dysbiosis noted in the GIT could not be a consequence of systemic inflammation following pulmonary fungal infection.

The immune system is the most studied component of the crosstalk between the lungs and the gut (21, 22, 47), and our results indicate that bacterial dysbiosis in the gut could be caused by the inflammation noted in this tissue. It can be assumed that T cells developed in response to the fungus, which have been shown to produce IFN-γ and IL-17 (51), migrate into the intestine and cause bacterial dysbiosis. Indeed, increased levels of IFN-γ (in the duodenum) and IL-17 (in all segments studied) were detected following pulmonary fungal infection, and obtained results are in agreement with our previous study in which inflammation was noted in the duodenum of rats with pulmonary infection caused by the fungus (33). This assumption is supported by the data indicating that dendritic cells from the lungs could increase the expression of gut homing receptors on T cells and induce the migration of cells to the gastrointestinal tract (53) as well as data regarding the migration of CD4^+^ T cells from the lungs to the intestine noted in pulmonary virus infection (21). Judging by the results obtained, inflammation was also present in the duodenum and is probably responsible for the decreased number of bacterial species in this segment of the intestine. The most interesting finding of the study was the presence of the IL-17 inflammation throughout the intestine during pulmonary infection. It should be noted that the IL-17 inflammation is probably limited to the mucosa, judging by the absence of changes in cytokine concentrations in plasma.

In contrast to the DA rat strain, no changes in bacterial number or composition were detected in feces of AO rats following pulmonary *A. fumigatus* infection. During pulmonary infection, the formation of fungus-specific cells that produce IFN-γ and IL-17 was documented in AO rats (34). The increased cytokine content in the GIT noted during infection and IL-17 detected in all examined segments of the intestine in AO rats indicate that migration of cells from the lungs to gut occurs in this rat strain as well. We hypothesize that intestinal bacterial composition in AO and DA rats might be responsible for the observed differences, as the fecal microbiota differs in healthy DA and AO rats. LEfSe analysis revealed a high number of genera that are differentially abundant in these strains. Judging by the obtained results, bacteria belonging to the class *Clostridia* have a higher relative abundance in AO than in DA rats, and this class of bacteria is known to affect the activity of regulatory T cells and to provide resistance to colitis and allergy (54). In agreement with these data, analysis of anti-inflammatory/immunoregulatory cytokine content revealed a higher value of IL-10 in healthy (uninfected) AO rats compared to DA, respectively, in the duodenum (295.0 ± 46.0 *vs* 230.0 ± 10.6, *P* = 0.0074), ileum (348.8 ± 35.2 *vs* 266.0 ± 36.5, *P* = 0.0104), cecum (431.3 ± 47.5 *vs* 364.0 ± 44.8, *P* = 0.0074), and colon (455.0 ± 14.1 *vs* 421.7 ± 23.5, *P* = 0.0100). As opposed to AO rats, in DA rats, LEfSe analysis pointed to higher abundance of representatives of the *Alphaproteobacteria*, *Betaproteobacteria,* and *Gammaproteobacteria* classes, whose relative abundance (or differential abundance determined by LEfSe) was shown to be higher in the small intestinal injury model (55) and the mouse model of colitis (56). In addition, treatment of old mice with mushroom polysaccharides that restore the immune function in aged animals (restore proinflammatory cytokine production) was shown to increase *Epsilonproteobacteria*, *Betaproteobacteria,* and *Deltaproteobacteria* (57). Taking into account all previously mentioned, it can be assumed that the different bacterial composition in the gut of AO and DA rats causes differences in basal immune tone in the gut (higher proinflammatory milieu in DA rats) that, following additional inflammatory stimulus, results in dysbiosis in DA but not in AO rats.

In general, the pulmonary infection caused by the fungus *A. fumigatus* has a differential effect on the bacterial community in the lungs and gut. While in the lungs increased alpha diversity and unchanged bacterial composition were noted, dysbiosis in the gut is characterized by decreased alpha diversity indices and altered bacterial composition. Lung–gut crosstalk might include some degree of migration of bacteria from the gut to the lungs and the immune system in the mucosal tissue. Furthermore, our results suggest that the alteration of the gut microbiota in response to pulmonary infection depends on the bacterial composition prior to infection. The data presented further support the existence of the lung–gut axis and provide additional insight into this mechanism.

## SUPPLEMENTAL MATERIAL

Supplemental figure and tables spectrum.01990-23-s0001.pdf DGGE profiles and peripheral blood parameters.

ASM does not own the copyrights to Supplemental Material that may be linked to, or accessed through, an article. The authors have granted ASM a non-exclusive, world-wide license to publish the Supplemental Material files. Please contact the corresponding author directly for reuse.
