# Supplementary material for: Gut microbial dysbiosis occurring during pulmonary fungal infection in rats is linked to inflammation and depends on healthy microbiota composition
Source: Microbiol Spectr. 2023 Aug 25;11(5):e01990-23. doi: 10.1128/spectrum.01990-23 (PMC10581041; doi:10.1128/spectrum.01990-23)
Supplement: Supplemental figure and tables — DGGE profiles and peripheral blood parameters. [file spectrum.01990-23-s0001.pdf]

Supplementary information

Gut microbial dysbiosis occurring during pulmonary fungal infection in rats is linked to inflammation and depends on healthy microbiota composition

Dusanka Popovic[1], Jelena Kulas[1], Dina Tucovic[1], Aleksandra Popov Aleksandrov[1], Anastasija Malesevic[1], Jasmina Glamoclija[2], Emilija Brdaric[3], Svetlana Sokovic Bajic[3], Natasa Golic[3], Ivana Mirkov[1], Maja Tolinacki[3,*]

[1] Immunotoxicology Group, Department of Ecology, Institute for Biological Research "Sinisa Stankovic" – National Institute of the Republic of Serbia, University of Belgrade, Belgrade, Serbia

[2] Mycology Laboratory, Department Plant Physiology, Institute for Biological Research "Sinisa Stankovic" – National Institute of the Republic of Serbia, University of Belgrade, Belgrade, Serbia

[3] Group for Probiotics and Microbiota-Host Interaction, Laboratory for Molecular Microbiology, Institute of Molecular Genetics and Genetic Engineering, University of Belgrade, Belgrade, Serbia

*Corresponding author. Email: maja_tolinacki@imgge.bg.ac.rs

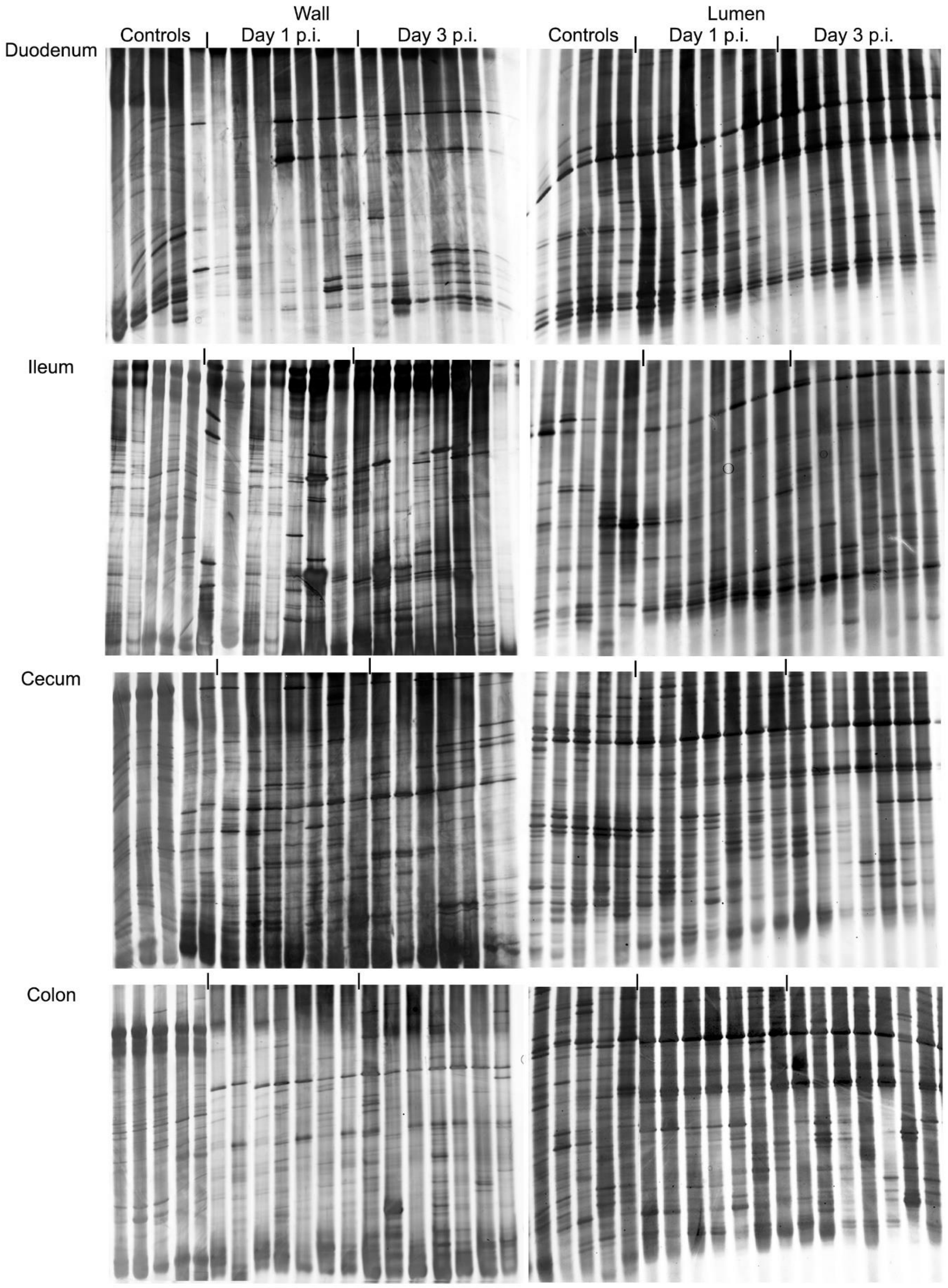

Fig. S1. Representative DGGE profiles of different segments in gastrointestinal tract.

Table S1. Number of DGGE bands in different segments of gastrointestinal tract.

	Controls	Day 1 p.i.	Day 3 p.i.
Duodenum			
wall	20.3 ± 2.7	12.5 ± 3.8***	18.0 ± 3.5
lumen	22.0 ± 2.0	22.0 ± 2.9	19.5 ± 2.8
Ileum			
wall	25.0 ± 2.9	22.0 ± 4.7	22.0 ± 5.5
lumen	21.0 ± 4.3	22.0 ± 3.2	19.0 ± 2.8
Cecum			
wall	22.0 ± 2.8	22.0 ± 4.2	18.0 ± 4.1
lumen	26.0 ± 4.7	28.0 ± 5.0	24.0 ± 4.4
Colon			
wall	20.5 ± 3.5	22.0 ± 3.7	20.0 ± 4.9
lumen (feces)	32.0 ± 5.0	25.5 ± 4.5***	20.5 ± 2.3***

Results are presented as mean ± standard deviation (SD) from 5-7 animals. Multiple comparisons between groups were done using one-way ANOVA followed by Tukey's test. Statistically significant differences at *** $p < 0.001$ for infected *vs.* control animals.

Table S2. Effect of pulmonary *Aspergillus fumigatus* infection on peripheral blood.

Parameter examined	Controls	Day 1 p.i.	Day 3 p.i.
Leukocytes (× 10^9/l)	10.3 ± 0.8	9.1 ± 1.1	8.7 ± 0.5
Lymphocytes (× 10^9/l)	5.8 ± 0.6	3.8 ± 0.6	3.6 ± 0.9
Neutrophil leukocytes (× 10^9/l)	3.5 ± 0.4	4.9 ± 0.9	4.8 ± 0.7
Monocytes (× 10^9/l)	0.2 ± 0.0	0.2 ± 0.0	0.2 ± 0.0
Eosinophil leukocytes (× 10^9/l)	0.09 ± 0.03	0.13 ± 0.06	0.11 ± 0.03
Basophil leukocytes (× 10^9/l)	0.06 ± 0.01	0.05 ± 0.01	0.03 ± 0.00
Red blood cells (× 10^{12}/l)	8.1 ± 0.3	8.1 ± 0.1	8.2 ± 0.1
Hemoglobin (g/l)	137.5 ± 2.8	137.4 ± 1.7	137.1 ± 1.7
Hematocrit (%)	0.45 ± 0.01	0.45 ± 0.01	0.44 ± 0.01
Platelets (× 10^9/l)	659.4 ± 37.3	615.5 ± 42.7	733.0 ± 19.9
Plasma IL-6 (pg/ml)	248.9 ± 25.4	266.7 ± 23.3	279.0 ± 22.6
Plasma TNF (pg/ml)	112.5 ± 11.8	107.5 ± 7.5	108.8 ± 6.9
Plasma IL-17(pg/ml)	35.1 ± 4.6	36.9 ± 2.4	31.0 ± 4.1

Results are presented as mean ± SD from eight animals per group. Multiple comparisons between groups were done using one-way ANOVA followed by Tukey's test.
